# Early Postnatal Heart Rate Variability in Healthy Newborn Infants

**DOI:** 10.3389/fphys.2019.00922

**Published:** 2019-08-07

**Authors:** Vânia Oliveira, Wilhelm von Rosenberg, Paolo Montaldo, Tricia Adjei, Josephine Mendoza, Vijayakumar Shivamurthappa, Danilo Mandic, Sudhin Thayyil

**Affiliations:** ^1^Centre for Perinatal Neuroscience, Imperial College London, London, United Kingdom; ^2^Department of Electrical and Electronic Engineering, Imperial College London, London, United Kingdom; ^3^Department of Neonatal Intensive Care, Università degli Studi della Campania Luigi Vanvitelli, Naples, Italy; ^4^Imperial College Healthcare NHS Trust, London, United Kingdom

**Keywords:** heart rate variability, healthy, term, infant, newborn

## Abstract

**Background:**

Despite the increasing interest in fetal and neonatal heart rate variability (HRV) analysis and its potential use as a tool for early disease stratification, no studies have previously described the normal trends of HRV in healthy babies during the first hours of postnatal life.

**Methods:**

We prospectively recruited 150 healthy babies from the postnatal ward and continuously recorded their electrocardiogram during the first 24 h after birth. Babies were included if born in good condition and stayed with their mother. Babies requiring any medication or treatment were excluded. Five-minute segments of the electrocardiogram (non-overlapping time-windows) with more than 90% consecutive good quality beats were included in the calculation of hourly medians and interquartile ranges to describe HRV trends over the first 24 h. We used multilevel mixed effects regression with auto-regressive covariance structure for all repeated measures analysis and *t*-tests to compare group differences. Non-normally distributed variables were log-transformed.

**Results:**

Nine out of 16 HRV metrics (including heart rate) changed significantly over the 24 h [Heart rate *p* < 0.01; Standard deviation of the NN intervals *p* = 0.01; Standard deviation of the Poincaré plot lengthwise *p* < 0.01; Cardiac sympathetic index (CSI) *p* < 0.01; Normalized high frequency power *p* = 0.03; Normalized low frequency power *p* < 0.01; Total power *p* < 0.01; HRV index *p* = 0.01; Parseval index *p* = 0.03], adjusted for relevant clinical variables. We observed an increase in several HRV metrics during the first 6 h followed by a gradual normalization by approximately 12 h of age. Between 6 and 12 h of age, only heart rate and the normalized low frequency power changed significantly, while between 12 and 18 h no metric, other than heart rate, changed significantly. Analysis with multilevel mixed effects regression analysis (multivariable) revealed that gestational age, reduced fetal movements, cardiotocography and maternal chronic or pregnancy induced illness were significant predictors of several HRV metrics.

**Conclusion:**

Heart rate variability changes significantly during the first day of life, particularly during the first 6 h. The significant correlations between HRV and clinical risk variables support the hypothesis that HRV is a good indicator of overall wellbeing of a baby and is sensitive to detect birth-related stress and monitor its resolution over time.

## Introduction

Heart rate variability (HRV) analysis provides insights into autonomic regulation and the interactions between sympathetic and parasympathetic nervous systems. HRV describes the variations in heart rate over time that occur naturally in healthy states. Those variations reflect the organism’s ability to continuously adjust to internal and external events, in order to maintain homeostasis. Interestingly, Chrousos and Gold (1992, p. 1245) defined stress as a “*state of threatened homeostasis*”. Therefore, over the years a decrease of HRV has been presumed to reflect an elevation in stress and HRV analysis has been increasingly recognized as one of the methodologies for measuring stress.

One of the simplest HRV measurements (SDNN) quantifies the standard deviation of the duration of normal RR intervals, i.e., how the interval between normal (sinus) R-peaks of consecutive QRS’s on the electrocardiogram (ECG) varies over time. Nonetheless, numerous mathematical metrics and approaches to HRV analysis have been developed over the years to extract more and more accurate information from HRV (Shaffer and Ginsberg, 2017).

Time domain HRV analysis is focused on the variation of the NN intervals (i.e., normal RR intervals) over time. In addition to the SDNN, HRV studies frequently examine the root mean square of successive differences (RMSSD) or the percentage of intervals that differ from the previous by more than 50 ms (pNN50) or 20 ms (pNN20). For all these HRV metrics in the time-domain, higher values reflect higher variability, which is more prevalent in healthy states.

Non-linear or geometric HRV analysis can be performed by plotting the NN-intervals on a Poincaré plot, where each NN interval is plotted in relation to the previous NN-interval (Golińska, 2013) and the standard deviation of the main cluster of data-points is measured crosswise (SD1) or lengthwise (SD2). Metrics such as the Cardiac Sympathetic Index (CSI) and Cardiac Vagal Index (CVI) have been developed to reflect the interactions between SD1 and SD2 (Toichi et al., 1997). CSI behaves in opposition to CVI, therefore, unlike most other HRV metrics, higher CSI is associated with lower variability, i.e., higher stress. Other geometric metrics include the Triangular Index (TINN) and the HRV Index. Like the SDNN, these two parameters indicate a measure of overall variability during the recording period. The TINN measures the normalized width of the base of histogram of the NN intervals (in relation to the highest value of the NN histogram) and the HRV Index is a ratio between the number of all NN intervals and the number of NN intervals at the highest point of the NN histogram (normalized to a sampling rate of 128 values per second).

In frequency domain analysis, different bands of the ECG power spectrum are analyzed as well as their interactions (between bands and in relation to the total power). In adults, previous studies have defined four frequency bands of interest: Ultra-low frequency (ULF), Very-low frequency (VLF), Low-frequency (LF), and High-frequency (HF), each of which is deemed to have different physiological origins. ULF has been associated with circadian oscillations of core body temperature and renin-angiotensin regulation; VLF has been associated with longer-term regulation of thermoregulation and hormonal mechanisms; LF has been associated with a mix of sympathetic and vagal activity and baroreceptor activity and HF has been associated with vagal activity (Pomeranz et al., 1985). Nonetheless, the definition and meaning of ULF and VLF in babies is under-documented and, therefore, is not included in this report. Although absolute quantifications of power in HF and LF bands can increase/decrease, under normal cardiac conductivity we expect LFn and HFn (which are LF and HF normalized to total power) to behave in opposite directions in most cases. Therefore, whereas HFn (representing parasympathetic activity) is expected to be higher when physiological stress is low, LFn is expected to be higher when stress is high. These associations are, nonetheless, controversial (von Rosenberg et al., 2017; Adjei et al., 2019) and the interpretation of LF and HF findings in real-life scenarios requires caution.

Heart rate variability analysis has been extensively accepted as a method to measure autonomic impairment and increasingly examined with regards to its value in disease stratification (Ahmad et al., 2009; Lees et al., 2018; Oliveira et al., 2018). Although previous studies have described normative HRV reference values for newborns in the first few days (Mehta et al., 2002; Longin et al., 2005; Doyle et al., 2009; Makarov et al., 2010; Lucchini et al., 2019), investigations into HRV during the initial hours after birth are lacking. These studies have mostly recorded only a few minutes of ECG during the first day or start only beyond 12 h of age and none have described continuous HRV trends during the first 24 h of life.

Nonetheless, for some conditions, particularly those occurring due to birth complications and requiring time-sensitive decisions, such as neonatal encephalopathy, it is important to describe normal HRV reference values immediately after birth and their trends throughout the first 24 h. Such early trends may provide valuable information on how a baby has recovered from any birth-related complication. Our primary aim was to describe standard reference values for HRV trends over the first 24 h of postnatal life in healthy term infants. As a secondary aim, we investigated which (if any) clinical characteristics or risk-factors exert higher impact on HRV.

## Materials and Methods

### Study Population

We prospectively and consecutively recruited 150 healthy term babies from the birth center, labor ward or postnatal ward at Queen Charlotte’s and Chelsea Hospital between August 2017 and January 2019. We included healthy babies born at 36 weeks gestational age or more, following uncomplicated pregnancies, who were born in good condition with a birth weight between the 9th and 91st centile. Babies were excluded if they required any medication or phototherapy, if there was perinatal maternal pyrexia during or within 48 h of the onset of labor, if they required resuscitation at or after birth (intubation or cardiac compressions or any drugs) or if there was any intrapartum complication (maternal hemorrhage, placental abruptio, pre-eclampsia or cord prolapse). Our study only included babies who were well at birth and, therefore, stayed with their mothers at all times.

### Intrapartum and Early Postnatal Care

Babies included in our study were born either at the birth center or in the labor ward, based on maternal preference. Women who preferred a more natural, less medicalized birth opted for midwife-led care in the birth center. A warm pool, aromatherapy, music, nitrous oxide and various equipment are available to help these women cope with the pain of labor. Women who opted for epidural analgesia received obstetrician-led care in the labor ward. In both environments, the room temperature was set at 24–25°C. As per national guidelines, babies born in good condition are given to the mother straight after birth and placed on their chest/abdomen for skin to skin care. They can be cleaned gently while on mother’s chest, and breast or bottle feeding initiated within 1 h of birth.

### ECG Acquisition

**FIGURE 1:**
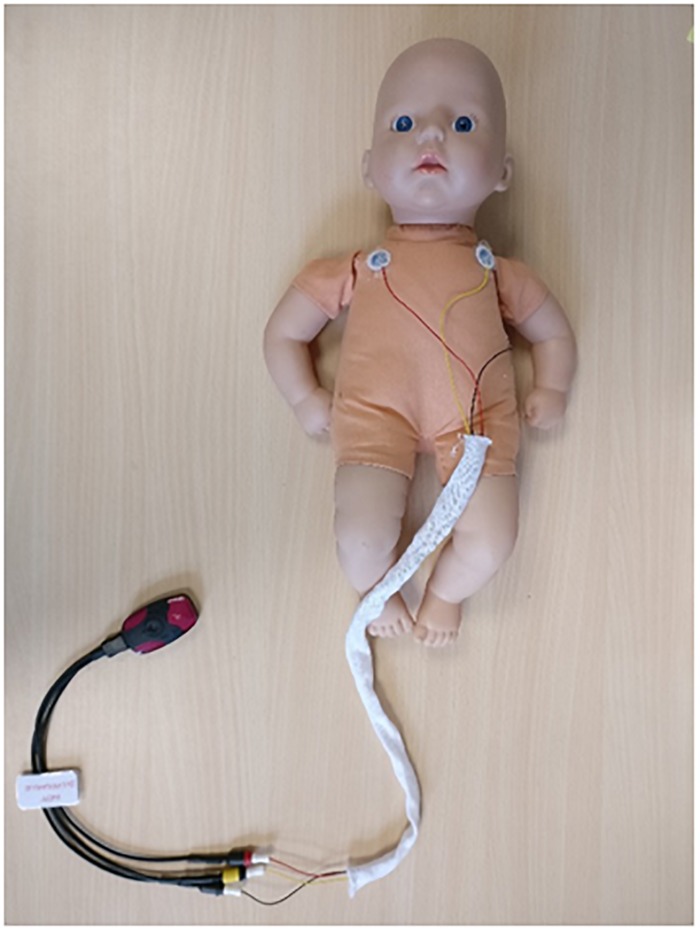
Portable ECG recorder and thoracic setup. Three ECG electrodes were fed through an alignment sleeve to reduce the distance between ECG wires and prevent magnetic induction artifacts on raw ECG signal (500 Hz with automatic R-peak detection).

Electrocardiogram recordings were started as soon as possible after birth, following parental informed written consent, which could be obtained antenatally or postnatally. This study was approved by a National Research Ethics Committee (REC17/LO/0956) and by the local Research & Development department. Recordings were continued for at least 6 h but could be interrupted earlier if requested by the parents or if the baby was being discharged. We used a portable 2-inch ECG recorder (Faros 180, Bittium, Oulu, Finland) with triple-electrode thoracic setup and a sampling rate of 500 Hz (Figure 1), which we had previously tested. Once the recording was completed, the ECG file was uploaded onto Cardiscope^TM^ HRV Analysis Software (Hasiba Medical, Graz, Austria) for ECG and HRV analysis.

### HRV Analysis

Heart rate variability metrics in the time (linear and non-linear) and frequency domains were calculated for each 5-min segment of the ECG (non-overlapping windows). We used a minimum QRS validity of 90%, which means all 5-min segments with less than 90% consecutive good quality QRS were excluded from analysis. Given the lack of international recommendations for HRV analysis specifically for neonates, our methods and chosen metrics were based on an adaptation of the available international guidelines (Task Force of the European Society of Cardiology and the North American Society of Pacing and Electrophysiology, 1996; Schwartz et al., 2002) plus recent recommendations for HRV research (Laborde et al., 2017) and a review of relevant HRV studies in neonates during the first days of life (Doyle et al., 2009; Goulding et al., 2015; Temko et al., 2015). The 16 HRV metrics we chose to analyze were based on these references. Frequency analysis was done with Fourier transform (Welch Periodogram) and we used detrended and interpolated (cubic spline) RR interval time series. Based on the above literature, we used a LF band of 0.04–0.20 Hz and a HF band of 0.20–2.0 Hz. We then analyzed normalized LF and HF, i.e., the proportion of power in those ranges in relation to the total spectral power. The list of HRV metrics reported in this study and their meaning are described in Table 1.

**TABLE 1:** HRV metrics: abbreviations and meaning.

**Metric**	**Definition**
HR	Heart rate (number of heart beats per minute)
**Time domain**	
SDNN	Standard deviation of the consecutive RR intervals
RMSSD	Root mean square of the consecutive RR differences
SDSD	Standard deviation of consecutive RR differences
(^∗^) pNN20	Proportion of NN intervals that differ from the previous interval by at least 20 ms
(^∗^) pNN50	Proportion of NN intervals that differ from the previous interval by at least 50 ms
**Geometric/Non-linear**	
SD1	Standard deviation of the Poincaré cross-wise
SD2	Standard deviation of the Poincaré lengthwise
CVI	Cardiac Vagal Index = log (SD1 × SD2)
CSI	Cardiac Sympathetic Index = SD1/SD2
TINN	Triangular Index of the NN intervals – the length of the basis of the minimum square difference of the triangular interpolation for the highest value of the RR histogram or the normalized width of the base of the RR histogram.
HRV Index	Number of all RR intervals divided by the number of RR intervals at the highest point of the RR histogram
Parseval Index	Ratio between the square root of the sum of LF and HF powers and the value of SDNN
**Frequency domain**	
HFn	Normalized power in the high frequency band of the ECG spectrogram (0.20–2.00 Hz), i.e., high frequency power in relation to total power
LFn	Normalized power in the low frequency band on the ECG spectrogram (0.04–0.20 Hz), i.e., low frequency power in relation to total power
Total power	Total power of the ECG spectrogram

*(^∗^) pNN50 has been widely used in adults but may be less relevant in neonates given their higher heart rate. Hence, we have also reported pNN20 to adjust for neonatal heart rate which corresponds to a 4–5% variation, in a heart rate of 120–160 bpm.*

### Statistical Analysis

We used Stata 15 (StataCorp, Austin, TX, United States) for the statistical analysis. We described HRV time trends with hourly medians and inter-quartile ranges and calculated the individual averages for the first 6 and 24 h of life. Since the ECG recordings could start and end at different times, our data was imbalanced, i.e., we did not have the exact same number of measurements for all participants, changes in HRV over time were analyzed with multi-level mixed effects regression with autoregressive covariance or using pairwise tests if comparing six hourly averages. The relevance of clinical variables was also tested with multi-level mixed effects regression with autoregressive covariance and subgroup comparisons were performed using proportions/mean comparison-tests for the significant clinical variables. As most trends were not linear and had at least one deflexion, we used a quadratic term for the time variable in the regression model. We log-transformed HRV variables which were not normally distributed to ensure normal residuals.

## Results

**FIGURE 2:**
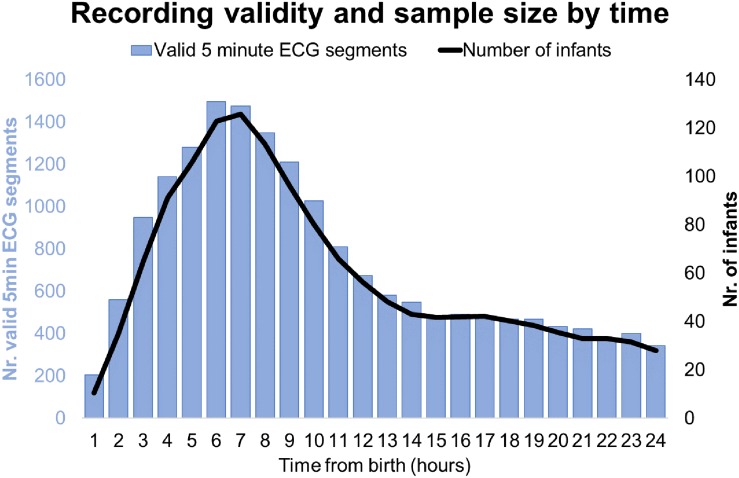
Recording validity and sample size by time.

Between September 2017 and January 2019, we screened 511 babies of which 360 were eligible and 151 were not. Out of those 360 babies, 201 mothers/fathers requested us to return later or at another convenient time, which eventually surpassed the maximum recruitment window or became impossible to recruit due to equipment being in use. Out of 159 mothers/fathers who were fully informed about the study, 9 declined and 150 gave informed written consent. Of these 150 participants, 7 started ECG recording after 24 h. Sample characteristics are reported in Tables 2, 3. In total, we obtained 1858 h and 55 min of ECG recording starting at a median (IQR) age of 2 h 46 min (3 h 6 min), minimum 1 min after birth, maximum 52 h 23 min. Not all babies started ECG recording at the same time nor did all recordings last the same duration. Figure 2 presents the number of valid recordings and babies by time.

**TABLE 2:** Sample characteristics I (continuous variables).

	**Mean(*SD*)**	**Min**	**Max**
Gestational age (weeks)	38.7 (1.26)	36	42
Birth weight (grams)	3267.6 (461.62)	2080	4670
Gravida	2.83 (1.65)	1	8
Para	1.29 (1.19)	0	7
Cord pH	7.26 (0.08)	6.99	7.50
Cord BE	−3.61 (3.44)	−15.0	2.2

*Gravida, number of pregnancies (including current); Para, number of previous live births; BE, base excess; Std, standard deviation; Min, minimum; Max, maximum.*

**TABLE 3:** Sample characteristics II (categorical variables).

**Variable**	**Category**	**Frequency(%)**
Single pregnancy	Yes	139 (92.7%)
	No	11 (7.3%)
TORCHS/Serology positive	Yes	3 (2%)
	No	147 (98%)
Smoking during pregnancy	Yes	2 (1.3%)
	No	148 (98.7%)
Alcohol during pregnancy	Yes	2 (1.3%)
	No	148 (98.7%)
Drugs during pregnancy	Yes	1 (0.7%)
	No	149 (99.3%)
Maternal depression	Yes	6 (4%)
	No	144 (96%)
Pregnancy induced or chronic maternal illness	Yes	46 (30.7%)
	No	104 (69.3%)
	Diabetes	22 (14.7%)
	Cardiac disease	0 (0.0%)
	Thyroid disease Epilepsy	6 (4%) 1 (0.7%)
	Hypertension	3 (2%)
	Other (^∗^)	20 (13.3%)
Medications during pregnancy	Yes	25 (17%)
	Unknown	5 (3%)
	No	120 (80%)
	Antibiotics	2 (1.3%)
	LMW heparin	2 (1.3%)
	Other (^∗∗^)	23 (15.3%)
Reduced fetal movements	Yes	12 (8%)
	No	138 (92%)
Cardiotocography	Not done	4 (2.7%)
	Unknown	1 (0.7%)
	Normal	132 (88%)
	Late deceleration	2 (1.3%)
	Bradycardia	2 (1.3%)
	Tachycardia	2 (1.3%)
	Variable deceleration	4 (2.7%)
	Other	3 (2.0%)
Analgesia during labor	No analgesia	9 (6%)
	50% Nitrous Oxide	40 (26.7%)
	General anesthesia	0 (0%)
	Spinal	1 (0.7%)
	Epidural	15 (10%)
	Combined Spinal and Epidural	86 (57.3%)
Labor onset	Spontaneous	53 (35.3%)
	Induced	28 (18.7%)
	Cesarean section (not in labor)	69 (46%)
Delivery mode	SVD	60 (40%)
	Forceps	2 (1.3%)
	Ventouse	12 (8%)
	Emergency Caesarean Section (not in labor)	1 (0.7%)
	Emergency Caesarean Section (in labor)	4 (2.7%)
	Elective Caesarean Section	71 (47.3%)
Labor and delivery events	No events	138 (92%)
	Antepartum hemorrhage	0 (0%)
	Shoulder dystocia	1 (0.7%)
	Meconium	11 (7.3%)
	Cord prolapse	0 (0%)
	Head entrapment	0 (0%)
	Other (^∗∗∗^)	2 (1.3%)
Any resuscitation at birth	No	145 (96.7%)
	Yes	5 (3.3%)
	Stimulation	2 (1.3%)
	Suction	2 (1.3%)
	Bag and mask/Intermitent positive pressure ventilation (IPPV)	1 (0.7%)
Apgar scores [Med (IQR)]	At 1 min	9 (0)
	At 5 min	10 (0)
	At 20 min	10 (0)
Feeding method during recording period	Breastfeeding	125 (83.3%)
	Bottle-feeding	25 (16.7%)

*TORCH – Toxoplasmosis, Rubella, Cytomegalovirus, Herpes simplex. Serology refers to any other available serology tests including Human Immunodeficiency Virus (HIV), Hepatitis B/C and Syphilis. (^∗^) Other pregnancy induced or chronic maternal illness: Asthma (7); Crohn’s disease (1); Hemophilia (1); Latent tuberculosis (1); Infertility problems (1); Group B streptococcus (1); Graves’ disease (1); Thalassemia carrier (1); Obstetric cholestasis (1); Hashimoto’s disease (2); Polycystic ovarian syndrome; Sickle cell carrier (1); Osteoporosis (1); Arthritis (1). (^∗∗^) Other Medications during pregnancy: Metformin (11); Dihydrocodeine (1); Insulin (2); Propranolol (1); Levothyroxin (4); Tinzaparin (1); Citalopram (1); Ursodeoxicolic acid (1); Levatiracetam (1) Amitriptyline (1); Salbutamol (2). (^∗∗∗^) Other labor and delivery events: Circular cervical cord (1); Prolonged 2nd stage of labor (1).*

### HRV Values Over Time

**FIGURE 3:**
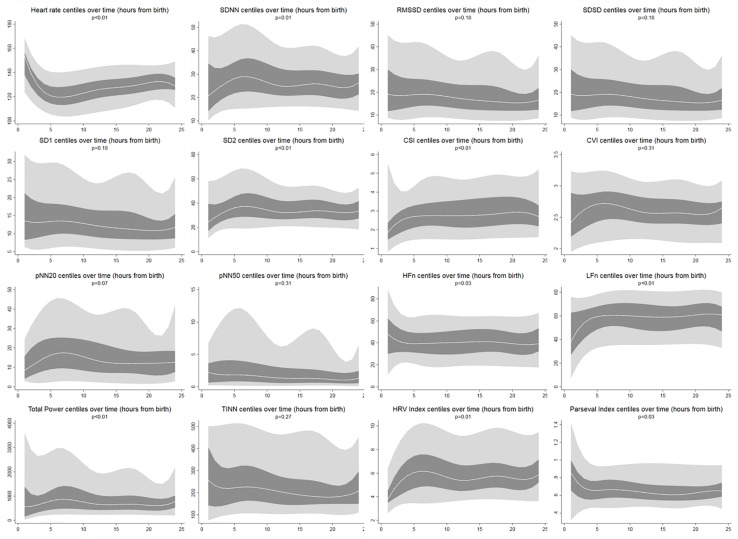
HRV centiles during the first 24 h of age. Light gray areas: 5th–25th centiles and 75th–95th centiles. Dark gray areas: 25th–75th centiles. Middle white line corresponds to the median. Reported *p*-values result from multi-level mixed effects regression to measure change over time [autoregressive covariance (time), repeated measures (id)]. All centiles smoothed with cubic spline.

**FIGURE 4:**
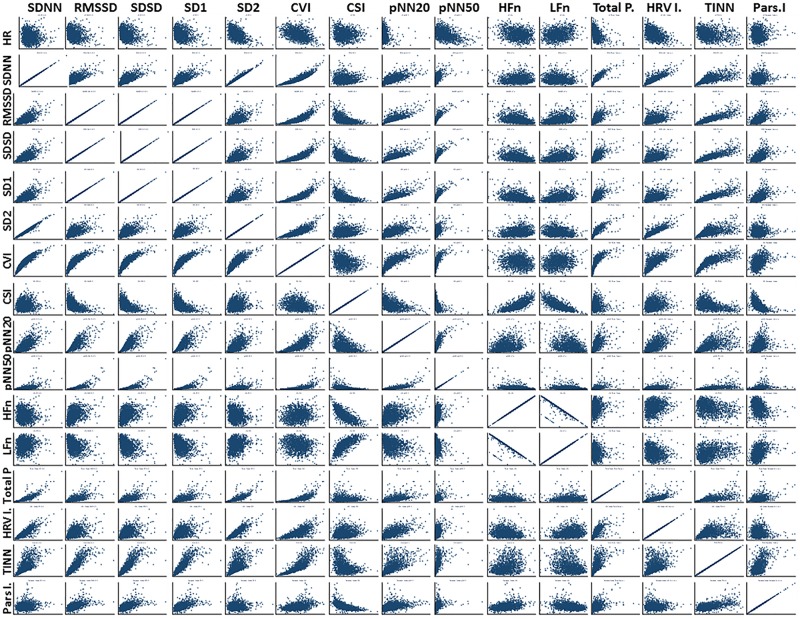
Correlation matrix: all HRV metrics. For abbreviations of HRV metrics, please refer to Table 1. We note particularly (a) the concordance between RMSSD, SDSD, and SD1, which has been previously described; (b) the behavior of different HRV metrics in relation to HR which highlights how much additional information HRV analysis can provide; (c) the interdependency of HFn and LFn and CVI and CSI, highlighting the interplay between sympathetic and parasympathetic influences.

During the first 6 h of postnatal age, median (IQR) values were HR 122 (15.9), SDNN 27.5 (13.2), RMSSD 18.32 (11.42), SD1 13.6 (7.7), SD2 36.2 (17.8), SDSD 18.8 (11.4), CVI 2.7 (0.4), CSI 2.6 (1.2), pNN20 14.8 (15.2), pNN50 1.7 (2.5) HFn 40.4 (16.9) LFn 57.7 (17.8) Total power 751 (835), TINN 226 (144) HRV Index 5.9 (2.3) Parseval 0.7 (0.2). Nine of these HRV metrics (including heart rate) changed significantly over time (HR *p* < 0.01; SDNN *p* = 0.01; SD2 *p* < 0.01; CSI *p* < 0.01; HFn *p* = 0.03; LFn *p* < 0.01;Total power *p* < 0.01; HRV Index *p* = 0.01; Parseval Index *p* = 0.03), adjusted for relevant clinical variables. A more pronounced variation was observed during the first 6 h of postnatal life. Only heart rate and LFn changed between 6 and 12 h of age and only heart rate changed between 12 and 18 h of age (Bonferroni adjusted *p*-values: 0.03, < 0.01 and < 0.01, respectively). These metrics exhibited increasing HRV during the first 6 h followed by a slight decrease up to 12 h from which point HRV remained stable (Table 4). Hourly trends expressed by median and interquartile ranges are presented in Figure 3. HRV trends over time were independently affected by gestational age [7 metrics: RMSSD (*p* = 0.01), SDSD (*p* = 0.01), SD1 (*p* = 0.01), CVI (*p* = 0.02), pNN20 (*p* = 0.03), TINN (*p* = 0.02), Parseval Index (*p* = 0.01)], reduced fetal movements [7 metrics: RMSSD (*p* = 0.01), SDSD (*p* = 0.01), SD1 (*p* = 0.01), CSI (*p* = 0.01), pNN20 (*p* = 0.01), pNN50 (*p* = 0.01), Parseval Index (*p* = 0.03)], cardiotocography (CTG) classification [3 metrics: CSI (*p* = 0.03), LFn (*p* = 0.01), HFn (*p* = 0.02)], maternal chronic or pregnancy induced illness [2 metrics: CSI (*p* = 0.02), Parseval Index (*p* = 0.01)] and occurrence of delivery complications [2 metrics: heart rate (*p* = 0.01) and CSI (*p* = 0.04)]. We examined the interdependencies of all HRV metrics with a correlation matrix (Figure 4), where we highlight (a) the similarity between time-domain metrics and (b) the trend toward symmetry between HFn and LFn and CSI and CVI.

**TABLE 4:** Six-hourly HRV averages and pairwise comparisons.

						**Overall**	**Pairwise comparison**		
**HRV Metric**		**6 h**	**12 h**	**18 h**	**24 h**	**24 h trend**	**6 vs. 12 h**	**12 vs. 18 h**	**18 vs. 24 h**
**HR**	Mean	124.05	121.66	125.92	130.47	*p* < 0.01	0.01	0.0018	0.25
	SD	0.96	0.83	1.35	1.16				
**SDNN**	Mean	29.81	29.56	27.32	26.45	*p* = 0.01	0.72	0.97	0.87
	SD	0.87	0.92	1.02	1.04				
**RMSSD**	Mean	21.78	21.08	19.18	18.58	*p* = 0.18	0.42	0.83	0.46
	SD	0.84	0.8	1.19	1.39				
**SD1**	Mean	15.4	14.9	13.56	13.14	*p* = 0.18	0.42	0.82	0.46
	SD	0.59	0.56	0.84	0.98				
**SD2**	Mean	38.48	38.41	35.44	34.39	*p* < 0.01	0.49	0.95	0.06
	SD	1.15	1.23	1.28	1.27				
**SDSD**	Mean	21.77	21.08	19.18	18.58	*p* = 0.18	0.42	0.82	0.46
	SD	0.84	0.8	1.19	1.39				
**CVI**	Mean	2.69	2.68	2.61	2.6	*p* = 0.31	0.07	0.93	0.47
	SD	0.03	0.03	0.04	0.04				
**CSI**	Mean	2.72	2.8	2.96	2.96	*p* < 0.01	0.10	0.90	0.72
	SD	0.07	0.07	0.14	0.14				
**pNN20**	Mean	18.33	19.66	16.21	15.01	*p* = 0.07	0.06	0.51	0.49
	SD	0.96	1.03	1.59	1.81				
**pNN50**	Mean	3.36	2.88	2.38	2.5	*p* = 0.31	0.23	0.25	0.94
	SD	0.4	0.31	0.37	0.79				
**HFn**	Mean	40.19	40.84	41.19	40.56	*p* = 0.03	0.67	0.90	0.77
	SD	1.1	1	1.8	1.68				
**LFn**	Mean	55.15	59.15	58.81	59.29	*p* < 0.01	0.0004	0.90	0.77
	SD	1.17	1	1.8	1.64				
**Total p.**	Mean	1098.2	1073	884.85	831.68	*p* < 0.01	0.31	0.69	0.34
	SD	94.43	81.96	78.09	76.98				
**TINN**	Mean	259.32	254.06	232.16	228.29	*p* = 0.27	0.88	0.84	0.17
	SD	8.94	9.35	15.4	1463				
**HRV Index**	Mean	6.17	6.2	6.01	5.95	*p* = 0.01	0.53	0.55	0.74
	SD	0.14	0.17	0.21	0.21				
**Parseval I.**	Mean	0.66	0.7	0.67	0.68	*p* = 0.03	0.04	0.99	0.09
	SD	0.01	0.01	0.02	0.02				

*Overall trend *p*-values result from multilevel mixed effects regression analysis, adjusted to all relevant clinical variables. Pairwise comparison *p*-values displayed are un-corrected for multiple comparisons. Bonferroni-corrected *p*-values for significant results are reported in the main body of text in the results section. For abbreviations of HRV metrics, please refer to Table 1.*

### Effect of Clinical Factors

In addition to age (time form birth), univariable multi-level mixed effects regression showed that HRV metrics were affected by gestational age, reduced fetal movements, CTG, maternal illness and delivery complications. After adjusting for these variables, the variation over time of the above seven HRV metrics remained statistically significant. HFn and Parseval Index also showed significant changes. Although parity was not an independent predictor on univariable regression analysis, in the subgroup of spontaneous vaginal deliveries, we observed a consistent trend toward higher HRV for primiparas in relation to multiparas.

#### Reduced Fetal Movements

**FIGURE 5:**
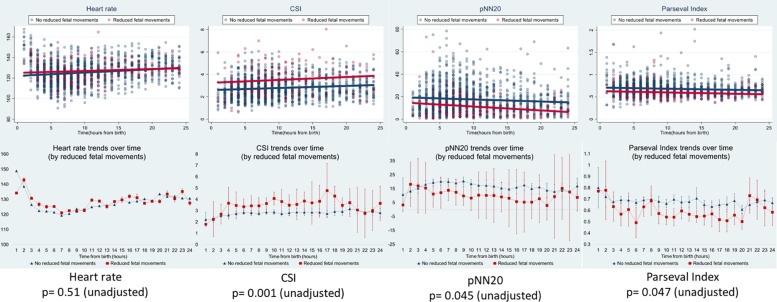
HRV changes in babies whose mothers reported reduced fetal movements. For abbreviations of HRV metrics, please refer to Table 1. Upper panels present HRV data scatter over time and linear trend lines for heart rate (comparative) and the three HRV metrics that were significantly associated with maternal reports of reduced fetal movements. Red lines represent babies whose mothers reported reduced fetal movements and blue lines represent babies whose mothers did not. The lower panels present the means and 95% confidence intervals for those metrics.

Reduced fetal movements were consistently associated with lower HRV values for all the metrics except for CSI (which behaves in the opposite direction, i.e., the result is concordant). Such differences between subgroups were only statistically significant for CSI (*p* = 0.001), pNN20 (*p* = 0.045) and Parseval Index (*p* = 0.047) trends. Figure 5 presents the trends of these three metrics over time, in comparison to heart rate. This is despite no significant differences between sub-groups, other than average gravidity, which did not independently predict HRV (Table 5). The associations with CSI and pNN20 remained significant after adjusting for gestational age, time from birth, CTG classification, presence of maternal chronic illness or delivery complications (Table 6). The subgroup with reduced fetal movements had lower average HRV during the first 6 h, although these differences were not statistically significant.

**TABLE 5:** Subgroup comparison – babies with and without reduced fetal movements.

**Clinical variable**	**No reduced fetal movements (*n* = 136)**	**Reduced fetal movements (*n* = 14)**	***p*-value**
Gestational age (weeks)	38.68	38.86	0.70
Birth weight (grams)	3266.47	3278.57	0.92
Cord base excess	−3.53	−4.14	0.61
Cord pH	7.26	7.25	0.71
Gravida	2.93	1.93	0.01
Para	1.34	0.86	0.16
Apgar 1 min	9 (0)	9 (0)	0.13
Apgar 5 min	10 (0)	10 (0)	0.18
Multiple Pregnancy	10 (7.3%)	1 (7.1%)	0.73
Positive TORCHS/Serology	4 (2.9%)	0 (0%)	0.67
Maternal smoking	2 (1.5%)	0 (0%)	0.82
Maternal alcohol use	2 (1.5%)	0 (0%)	0.82
Maternal drug use	1 (0.7%)	0 (0%)	0.91
Maternal depression	6 (4.4%)	0 (0%)	0.55

*Values presented are in means for variables Gestational age to Para; medians (inter-quartile ranges) for Apgar scores and proportions (percentages) for remaining variables. Gravida – number of previous pregnancies including the one leading to current baby. Para – number of previous births. TORCH – Toxoplasmosis, Rubella, cytomegalovirus, Herpes simplex. Serology refers to any other available serology tests including Human Immunodeficiency Virus (HIV), Hepatitis B/C, and Syphilis. *P*-values derived from unequal variances *t*-test for continuous variables and Fisher’s exact test for binary variables.*

**TABLE 6:** Unadjusted and adjusted correlation between HRV metrics and reduced fetal movements.

	**Unadjusted**					**Adjusted**				
	**Coef.**	***SD***	***p***	**95% CI**		**Coef.**	***SD***	***p***	**95% CI**	
CSI	0.66	0.21	0.011	0.26	1.07	0.65	0.22	0.003	0.22	1.08
pNN20	−5.96	2.98	0.045	−11.8	−0.13	−7.53	3.24	0.02	−13.9	−1.17
Parseval	−0.07	0.36	0.047	−0.14	−0.00	−0.08	0.4	0.054	−0.15	0.00

*For abbreviations of HRV metrics, please refer to Table 1. Analysis based on multi-level mixed effects regression analysis (auto-regressive covariance). Adjusted results based on including all clinical variables that were significant predictors on univariable analysis, i.e., gestational age, time from birth, CTG, maternal illness, and delivery complications.*

#### Cardiotocography

There was a significant association between CTG findings and HRV trends over time for CSI (*p* = 0.03), LFn (0.01), and HFn (*p* = 0.02), which remained statistically significant after adjusting for reduced fetal movements, gestational age, time from birth, presence of chronic maternal or pregnancy induced illness or delivery complications. Nonetheless, given the small number of events in each CTG classification we also compared the 6 h mean across these subgroups. CSI in the bradycardia subgroup was significantly different from the “normal,” “variable deceleration,” and “other” groups (Bonferroni adjusted *p*-values: 0.02, 0.03, and 0.04, respectively) but no other group differences were statistically significant. HFn was only different between “bradycardia” and “variable deceleration” subgroups (*p* = 0.03) and LFn did not vary between different CTG subgroups.

#### Maternal Chronic or Pregnancy Acquired Illness

In our sample, 104 (69%) women had no chronic illness nor pregnancy induced disease, 20 (13%) had isolated diabetes mellitus or gestational diabetes, 7 (3%) had thyroid disease, 1 (0.7%) had hypertension and the remaining [22 (15%)] had other conditions, including combinations of the above (Table 3). Maternal chronic or pregnancy induced illness was significantly associated with unadjusted HRV trends [CSI (*p* = 0.02) and Parseval Index (*p* = 0.01)] although this only remained statistically significant for Parseval Index (*p* = 0.03) after adjusting for clinical confounders. On binary analysis, babies of mothers with pregnancy/chronic illness did not have different average Parseval Indexes either during the first 6 h (*p* = 0.98) or during the 24 h period (*p* = 0.29). The disease group with lowest Parseval Index was thyroid disease.

#### Events During Labor and Delivery

All babies in our study were born following uncomplicated pregnancies and deliveries and born in good condition. Nonetheless, there were 13 births that had one of the following events: meconium stained liquor (11), circular cervical cord (1), prolonged 2nd stage of labor (1) and shoulder dystocia (1, in addition to meconium). CSI trends over 24 h were significantly associated with presence of any labor and delivery events before (*p* = 0.04) but not after adjusting for confounders (*p* = 0.42).

Although delivery mode did not independently predict HRV values, babies born via instrumental delivery had lower 24 h HFn and higher 24 h CSI [mean (95% CI) 36.7 (34.0–39.4) vs. 42.3 (41.2–43.3) and 3.1 (2.9–3.3) vs. 2.7 (2.7–2.8), respectively, *p* = 0.002] than babies born via natural vaginal delivery. This was despite no significant differences between other relevant clinical variables, except Apgar Score at 1-min and multiplicity of gestation which did not independently predict HRV (Table 7).

**TABLE 7:** Subgroup comparison – babies born via normal vaginal versus instrumental delivery.

**Clinical variable**	**Spontaneous vaginal delivery (*n* = 60)**	**Instrumental delivery (*n* = 14)**	***p*-value**
Gestational age (weeks)	38.77	37.89	0.45
Birth weight (grams)	3242.69	2955.56	0.39
Cord base excess	−4.54	−5.57	0.47
Cord pH	7.25	7.20	0.06
Gravida	3.27	2.11	0.39
Para	1.69	0.55	0.08
Apgar 1 min	9 (0)	9 (1)	0.03
Apgar 5 min	10 (0)	10 (0)	0.52
Reduced fetal movements	6 (10%)	3 (21.4%)	0.22
Multiple Pregnancy	0 (0%)	2 (14.3%)	0.03
Positive TORCHS/Serology	0 (0%)	0 (0%)	Not applicable
Maternal smoking	1 (1.7%)	0 (0%)	0.81
Maternal alcohol use	0 (0%)	0 (0%)	Not applicable
Maternal drug use	0 (0%)	1 (7.1%)	0.19
Maternal depression	2 (3.3%)	2 (14.3%)	0.16

*Values presented are in means for variables Gestational age to Para; medians (inter-quartile ranges) for Apgar scores and proportions (percentages) for remaining variables. Gravida – number of previous pregnancies including the one leading to current baby. Para – number of previous births. TORCH – Toxoplasmosis, Rubella, cytomegalovirus, Herpes simplex. Serology refers to any other available serology tests including Human Immunodeficiency Virus (HIV), Hepatitis B/C, and Syphilis. *P*-values derived from unequal variances *t*-test for continuous variables, Krushkal–Wallis for non-parametric variables and Fisher’s exact test for binary variables.*

## Discussion

This is the first study to describe early postnatal continuous HRV trends in healthy term babies in the immediate postnatal period. Identifying these thresholds and trends was important because we now know that HRV analysis and interpretation in the early postnatal period is time-dependent, i.e., what is normal at 1–6 h of age may not be normal at 12–18 h of age. This will allow clinicians and researchers to more accurately examine the differences in HRV between healthy and sick infants in the immediate postnatal period. Having accurate reference values for the immediate postnatal period also means that we are now better equipped to develop early warning systems based on HRV analysis.

There are some possible reasons for the changes we observed in the first 6 h after birth. Since birth has been previously described as a stressful event for babies (Peebles et al., 1994; Aldrich et al., 1996), it is possible that the improving HRV in the first hours reflects the end of the stressor effect (i.e., the end of birth). This parasympathetic rebound could occur because the sympathetic nervous system can temporarily supress parasympathetic activity which stops once the stress period is over. On a different perspective, Reyes-Lagos et al., 2015 reported higher maternal HRV during labor than during the third trimester and Musa et al., 2017 described increasing LFn and HFn by cervical dilation during labor. If fetal HRV follows maternal HRV, this would instead suggest that birth may represent a period of particularly high HRV reflecting the good adaptation to physiological challenges, in healthy babies. Finally, it is also possible that the changes in HRV observed during the first hours are a partial expression of the HRV maturation that occurs with age (Fyfe et al., 2015).

We have reviewed HRV findings reported in other neonatal and fetal studies with the intent of comparing such values with those in our study but given the differences in metrics and acquisition and duration for recordings, this was very challenging. The HRV values we observed in the first 6–12 h were comparable (slightly higher) to those reported by Doyle et al. (2009) in active sleep during the first 12 h of life and Lucchini et al., 2019 at 12–84-hour-old and lower than those reported at older ages, i.e., 24–168 h (by Mehta et al., 2002; Longin et al., 2005; Makarov et al., 2010). Babies in our study had SDNN values during the first 6 h of age that were comparable (slightly higher) to those in term fetuses (Brändle et al., 2015; Schneider et al., 2018), which was expected, given the longer recording duration and increasing gestational age. Having similar HRV before and after birth supports the theory that birth, in healthy babies, is associated with good HRV stability.

We observed more inter-subject than within-subject variability across all HRV metrics. This emphasizes that HRV analysis must be interpreted based not only on reference values but also take into consideration the changes and progress in relation to individual baselines. In fact, whereas we could observe hourly HRV changes during the first 6 h of age, we might not have been able to detect such variation if we had only committed to analyze a single time-point or average. Monitoring such trends may provide further insight about how an infant recovers from birth in the event of a complicated delivery or emergency intervention. Indeed, the fact that HRV metrics were significantly associated with reduced fetal movements, abnormal CTG, maternal chronic or pregnancy induced illness and delivery complications highlights the value of HRV analysis as a measure of overall wellbeing.

The association between HRV and fetal movements has been previously reported by Brändle et al. (2015) using fetal biomagnetography. In their study assessing behavior state based on movements which included healthy fetuses from 24 to 41 weeks, HRV metrics (but not Entropy) were increasing from quiet sleep to active sleep and from active sleep to awake state, for all gestational ages. In fact, Nijhuis et al. (1982) had already proposed a fetal movement classification based on fetal HRV, eye movement and body movement.

Previous authors have found differences in HRV values across different delivery modes. Kozar et al. (2018) reported lower HFn and higher LFn in babies born via cesarean section than in babies born vaginally. It is possible that their finding is related with the use of Thiopental for general anesthesia (GA) in all their elective sections (Riznyk et al., 2005; Tsuchiya et al., 2006) rather than with the delivery mode, whereas in our study there was no use of any GA. Our interpretation is that HRV will indicate a difference between delivery modes if there is a difference in wellbeing, therefore, in our study, this would have been associated with the use of instruments during delivery due to difficult extraction.

### Limitations

We have not examined the sleep states of the recruited infants during the first 24 h after birth. It is unlikely that babies would have established a circadian within few hours of birth and often newborn babies follow ultradian rhythms instead (Mirmiran et al., 2003). Nevertheless, it is possible that the slightly downward trend that we observed in the second half of the 24 h recordings reflects a bigger proportion of babies sleeping or in a quieter state. We have included a few healthy babies in our study whose mothers had some chronic or pregnancy induced illnesses. It could be argued that including these cases compromises our definition of healthy newborn and healthy neonatal HRV. The fact that there were no significant associations between any HRV metric and pH or base excess or Apgar score highlights that our sample was indeed healthy, as all the babies in our study were born in good condition and did not require any type of investigations or treatment. Our aim was to represent the whole spectrum of deliveries that are considered and clinically managed as “healthy” from a pragmatic point of view (i.e., all “low-risk” and “uncomplicated”). Thus, including those babies was an important step in addressing possible “variations of normal” and to enrich our dataset. Equally, we have explored possible differences in HRV across several subgroups (according to clinical variables such as described in Table 2). It is important to clarify that our study was not aimed at or powered to address in detail possible differences across those subgroups and caution is needed to interpret our findings. Nonetheless, we believe that examining these associations will be of interest for healthcare teams in obstetric and perinatal/neonatal care.

## Conclusion

We have described the early postnatal reference values for HRV metrics in healthy term infants, which had not been previously achieved. HRV changes significantly during the first day of life, particularly during the first 6 h, during which it seems to exhibit a brief increase followed by normalization. The significant correlations between HRV and clinical risk variables support the hypothesis that HRV is a good indicator of overall wellbeing of a baby and is sensitive to pick up birth-related stress and monitor its resolution over time.

## Data Availability

The raw data supporting the conclusions of this manuscript will be made available by the authors, without undue reservation, to any qualified researcher.

## Ethics Statement

This study was carried out in accordance with the recommendations of the United Kingdom Health Research Authority and the GCP ICH with written informed consent from all subjects. All subjects gave written informed consent in accordance with the Declaration of Helsinki. The protocol was approved by the London Chelsea Research Ethics Committee.

## Author Contributions

VO designed the study, collected, analyzed, and interpreted the data, wrote the first draft and led the development of the manuscript. WvR contributed to the analysis and interpretation of the data and provided critical inputs for developing the manuscript. PM and TA contributed to the interpretation of the data and provided critical inputs for developing the manuscript. JM contributed to the study design and data interpretation, recruited patients and acquired data. VS contributed to the study management and recruitment. DM supervised all aspects of HRV analysis and interpretation and provided critical inputs for developing the manuscript. ST conceived the idea and designed the study, supervised all aspects of the study and led the development of the manuscript.

## Conflict of Interest Statement

The authors declare that the research was conducted in the absence of any commercial or financial relationships that could be construed as a potential conflict of interest.
